# Depression as a Consequence of Presbycusis: A Study From a Government Tertiary Care Center in Uttarakhand

**DOI:** 10.7759/cureus.88912

**Published:** 2025-07-28

**Authors:** Gautam S Mourya, Vikas Sikarwar, Chettali Bhatt

**Affiliations:** 1 Otolaryngology - Head and Neck Surgery, Government Doon Medical College, Dehradun, IND

**Keywords:** age-related hearing loss, depression, geriatric health, hamilton depression rating scale, health outcomes in aging, hearing research, mental health, presbycusis, psychiatry of old age, psychosocial impact

## Abstract

Background

Presbycusis, also known as age-related hearing loss (ARHL), is one of the most prevalent sensory deficits among the elderly and is often associated with social isolation and cognitive decline. This study aimed to investigate the association between ARHL and depression in the geriatric population of Uttarakhand, India.

Methods

A prospective cross-sectional study was conducted between January 1, 2024, and February 29, 2024, at the Government Doon Medical College Hospital, Uttarakhand. Participants were recruited through convenience sampling from consecutive patients meeting the eligibility criteria during the study period. Presbycusis was confirmed using pure tone audiometry and speech audiometry. Depression was diagnosed by qualified psychiatrists according to the International Classification of Diseases 11th Revision (ICD-11) criteria and assessed using the Hamilton Depression Rating Scale (HDRS-17) under psychiatric supervision. Patients with comorbidities known to cause depression, a previous history of psychiatric disorders, conductive hearing loss, chronic suppurative otitis media, and otosclerosis, and those using hearing aids were excluded. Data analysis was performed using Stata software version 18.0 (StataCorp LLC, College Station, TX), with statistical tests including the Chi-square, Fisher's exact t-test, and one-way analysis of variance (ANOVA).

Results

The study included 65 non-institutionalized geriatric patients diagnosed with ARHL, with a mean age of 68.4 ± 7.86 years, of whom 61.5% were male patients. Among the participants, 36.8% had mild hearing loss, 40% had moderate hearing loss, 16.9% had severe hearing loss, and 6.2% had profound hearing loss. Depression was present in 89.2% of the study population, with 50.8% experiencing mild depressive symptoms, 26.2% moderate depression, and 12.3% severe depression. A statistically significant positive correlation was found between hearing loss and depression (r = 0.538, p = 0.000). Chi-square analysis further revealed a significant association between the severity of depressive symptoms and the degree of hearing loss (p = 0.001).

Conclusion

The study provides compelling evidence of a strong association between ARHL and depression in the elderly population of Uttarakhand. Moreover, the findings suggest that greater severity of hearing loss correlates with higher levels of depressive symptoms, underscoring the need for early diagnosis and intervention. Addressing ARHL not only improves hearing outcomes but may also reduce the risk of depression and enhance overall well-being in ageing populations. Further research is warranted to develop effective therapeutic strategies that integrate audiological and mental healthcare for this vulnerable demographic.

## Introduction

Presbycusis, or age-related hearing loss (ARHL), is one of the most prevalent sensory impairments among the elderly population [1,2]. Its multifactorial etiology is influenced by intrinsic and extrinsic factors [1]. Recent studies indicate that the prevalence of hearing loss is 25%-40% in individuals over 65 years old, 40%-66% in those over 75 years old, and 80%-90% in individuals above 85 years old [1]. Furthermore, emerging research has suggested a potential link between ARHL and cognitive decline, dementia, and depression in the elderly [2,3].

Hearing loss significantly affects communication, leading to social withdrawal and isolation, which are key contributors to depression [1]. With India projected to have the largest geriatric population by 2025 [4], addressing age-related health concerns has become increasingly critical. India's elderly population comprises 8.6% of the total population, with the state of Uttarakhand having a slightly higher proportion at 8.9% [4]. Despite the growing elderly population, mental health conditions such as depression remain underdiagnosed in this demographic [5]. The state's predominantly rural population, with limited access to specialized healthcare services, makes it particularly vulnerable to undiagnosed sensory impairments. Additionally, the cultural context of joint family systems in Uttarakhand may mask the social impact of hearing loss, as family support structures might compensate for communication difficulties.

According to the World Health Organization (WHO), depression is characterized by persistent sadness and a lack of interest in previously pleasurable activities. A meta-analysis estimates that the global prevalence of depressive symptoms in the elderly ranges between 4.7% and 16%, whereas in India, the prevalence is significantly higher at 21.9% [5]. Despite this concerning trend, psychiatric disorders in older adults remain underexplored, particularly when compared to other health conditions. The association between ARHL and depression has not been sufficiently investigated, especially in developing countries like India and, more specifically, in Uttarakhand. This study aims to establish evidence-based correlations between ARHL and depression in the geriatric population of Uttarakhand, a state in northern India.

## Materials and methods

This prospective cross-sectional study was conducted to assess the relationship between age-related hearing loss and depression in the elderly population. This study was conducted between January 1, 2024, and February 29, 2024, at the Department of Otorhinolaryngology in association with the Department of Psychiatry at Government Doon Medical College Hospital, Uttarakhand, India. This is a tertiary-level hospital affiliated with Hemwati Nandan Bahuguna Medical University.

Objective

This study aimed to assess the association of age-related hearing loss with depression in elderly patients and to determine the correlation between the severity of hearing loss and depression. This study also aimed to compare the severity of age-related hearing loss in the elderly by sociodemographic variables.

Study population and sampling

The study population comprised non-institutionalized geriatric patients diagnosed with age-related hearing loss who visited the Otorhinolaryngology Outpatient Department (OPD) of Government Doon Medical College Hospital. Participants were recruited through convenience sampling from consecutive patients meeting the eligibility criteria during the study period.

Inclusion and exclusion criteria

The study included patients aged 60 years [4] and above who were diagnosed with presbycusis, irrespective of gender. Patients were excluded if they had any of the following conditions: hypertension, diabetes mellitus, hypothyroidism, Parkinson's disease, dementia, or blindness. Additional exclusion criteria comprised any comorbid condition that could cause depressive disorder (as determined by clinical history and systemic examination), prior history of depressive disorders or psychiatric treatment, history of chronic suppurative otitis media (active or inactive), conductive or mixed hearing loss, and otosclerosis. Patients who were currently using hearing aids were excluded, as auditory compensation could potentially reduce depressive symptoms and confound the study results. Finally, patients who were unable to complete the required audiological or psychiatric assessments were excluded from the study.

Sample size

The sample size was calculated using Statulator, an online calculator for statistical analysis (https://statulator.com). Considering that 20% of the participants in the population have presbycusis [6], and an expected response rate of 95%, the following formula was used:

$$n = \frac{NX}{(X+N-1)}$$

where:

\[ X = \frac{ \ Z_{\alpha/2}^2 \cdot p \cdot (1-p)}{(MOE^2)} \]

Zα/2 is the critical value of the normal distribution at α/2, MOE is the margin of error, p is the sample proportion, and N is the population size [7]. Zα/2 = 1.96 (critical value of normal distribution at α/2 = 0.025). It was inferred that the study would require a sample size of 65 to estimate the expected proportions with 10% absolute precision and 95% confidence.

Data collection and data processing

Data were collected using validated, pre-approved instruments and questionnaires. Elderly patients visiting the Otorhinolaryngology clinic with a clinical history indicative of presbycusis underwent a comprehensive audiological evaluation. Before audiometric testing, otoscopic examination was performed to exclude external ear canal and tympanic membrane pathologies, and upper respiratory tract infections were also ruled out to ensure optimal testing conditions. All audiological assessments were conducted by a trained audiologist using standardized protocols. The hearing thresholds of the participants were assessed at frequencies of 250, 500, 1,000, 2,000, 3,000, 4,000, 6,000, and 8,000 Hz. It was recorded for each ear independently and for each frequency mentioned above. The thresholds were measured in decibels (dB), with 0 dB representing the average hearing ability of an adult with no ear pathology. Larger thresholds indicated poorer hearing outcomes. Speech audiometry was performed to assess functional hearing ability. Based on these comprehensive audiological findings, a diagnosis of presbycusis was established, with hearing loss severity classified according to the World Health Organization hearing impairment grading system [8]. Patients diagnosed with presbycusis were subsequently referred to the Psychiatry Department of Government Doon Medical College for a comprehensive psychiatric assessment conducted by a qualified psychiatrist following the International Classification of Diseases 11th Revision (ICD-11) guidelines to identify symptoms of depressive disorder. Patients meeting diagnostic criteria for depression underwent severity assessment using the Hamilton Depression Rating Scale (HDRS-17), a widely utilized clinician-administered scale that evaluates multiple domains including mood, guilt, suicidal ideation, insomnia, psychomotor symptoms, anxiety, weight loss, and somatic complaints, with scoring performed by summing the highest ratings across all 17 items and total scores ranging from 0 to 23 and above. Depression severity was categorized according to standardized score ranges: normal (0-7), mild depression (8-13), moderate depression (14-18), severe depression (19-23), and very severe depression (≥24) [9]. The level of depression was compared with the corresponding degrees of hearing loss. To minimize assessment bias, depression severity gradings were concealed from the psychiatrists conducting the evaluations, ensuring a blinded assessment of psychiatric symptoms relative to audiological findings.

Statistical analysis

The collected data were entered into Microsoft Excel (Microsoft Corp., Redmond, WA) and exported to Stata software version 18.0 (StataCorp LLC, College Station, TX) for statistical analysis. Before analysis, the normality of the data was checked using the K-S test. The data is expressed as mean ± standard deviation (SD) for normally distributed variables. Categorical variables were compared using either the Chi-square test or Fisher’s exact t-test, as appropriate. Continuous variables were analyzed using Student's t-test for unpaired data. Comparison between means across three or more groups was conducted using the one-way analysis of variance (ANOVA) procedure. Correlation between continuous variables was assessed using the Pearson correlation. All statistical tests were two-tailed, and a p-value < 0.05 was considered statistically significant [7].

Quality control

Audiological data quality control was maintained by regular audiometer calibration, ensuring that the hearing level complies with strict values set by the manufacturer's specific references for hearing thresholds. This ensured precise and accurate data.

Approval and confidentiality

This project was approved by the Institutional Ethics Committee of Government Doon Medical College and Hospital, Dehradun (reference number: GDMC/IEC/2023/90). This project gained approval from the Indian Council of Medical Research under the Short-Term Studentship Programme (ICMR-STS). The personal and medical details of all participants are kept confidential by the researcher and are limited to research purposes only.

## Results

The research was conducted to verify the existence of an association between the presence and degree of hearing loss and depression in non-institutionalized elderly.

Population characteristics

As shown in Table 1, a total of 65 patients, aged between 60 and 90 years, were evaluated, with a mean age of 68.4 ± 7.86 years. The study population comprised 40 (68.5%) male patients and 25 (38.5%) female patients, resulting in a sex ratio of 2.66. Among the participants, 10 (15.4%) consumed alcohol, while 55 (84.6%) were non-consumers. Similarly, 10 (15.4%) were tobacco users, whereas 55 (84.6%) were non-users. Regarding occupational noise exposure, 13 (20%) participants were employed in high-noise environments, while 52 (80%) participants worked in low-noise settings.

**Table 1:** Percentage and frequency of population characteristics High noise exposure: carpenter, army, and bus conductor Low noise exposure: homemaker, shopkeeper, clerk, electrician, and farmer

Variable	Subcategory	Frequency	Percentage
Sex	Male	40	61.5
	Female	25	38.5
Alcohol	Yes	10	15.4
	No	55	84.6
Tobacco	Yes	10	15.4
	No	55	84.6
Age	>70 years	31	47.7
	<70 years	34	52.3
Occupation	High noise exposure	13	20.0
	Low noise exposure	52	80.0

Hearing loss characteristics

As presented in Table 2, the severity of hearing loss among participants ranged from mild to profound. A total of 24 (36.8%) individuals had mild hearing loss, 26 (40%) had moderate hearing loss, 11 (16.9%) had severe hearing loss, and four (6.2%) had profound hearing loss.

**Table 2:** Presence and degree of hearing loss in the sample components (WHO grading of hearing loss) WHO: World Health Organization

Grade	Frequency (N = 65)	Percentage	Range (in sample)	Mean ± standard deviation
Mild hearing loss (25-40)	24	36.8	27-40	34.38 ± 4.282
Moderate hearing loss (41-60)	26	40	41-60	50.576 ± 5.884
Severe hearing loss (61-80)	11	16.9	61-80	68.8 ± 5.9814
Profound hearing loss (≥81)	4	6.2	83-98	88 ± 5.873

For mild hearing loss, the range was 27-40 decibels (dB), with a mean score of 34.38 ± 4.282 dB. In cases of moderate hearing loss, absolute scores ranged from 41 to 60 dB, with a mean score of 50.58 ± 5.88 dB. Severe hearing loss scores fell within the 61-80 dB range, with a mean score of 68.8 ± 5.98 dB. For profound hearing loss, scores ranged from 83 to 98 dB, with a mean score of 88 ± 5.87 dB.

Depression severity based on the Hamilton Depression Rating Scale

According to data extracted from the Hamilton Depression Rating Scale (Table 3), 58 (89.2%) participants exhibited depressive symptoms ranging from mild to severe, while seven (10.8%) participants showed no signs of depression. The majority of subjects (33 (50.8%)) had mild depression, followed by 17 (26.2%) participants with moderate depression, and eight (12.3%) participants with severe depression. Notably, no subject was found to have very severe depression.

**Table 3:** Presence and degree of depressive symptoms in the sample components

Grade	Frequency (N = 65)	Percentage	Range	Mean ± standard deviation
No (0-7)	7	10.8	3-7	5.28 ± 1.277
Mild (8-13)	33	50.8	8-13	10.09 ± 1.747
Moderate (14-18)	17	26.2	14-17	14.04 ± 0.52
Severe (18-23)	8	12.3	18-23	21.09 ± 2.50

Depression scores among the study population ranged from 3 to 23, with a mean score of 12.8 ± 6.18. In the "no depression" category, scores ranged from 3 to 7, with a mean score of 5.28 ± 1.28. For the "mild depression" category, scores ranged from 8 to 13, with a mean score of 10.09 ± 1.75. The "moderate depression" category had a mean score of 14.04 ± 0.52, with scores ranging from 14 to 18. Lastly, the "severe depression" category had the highest mean score of 21.09 ± 2.50, with scores ranging from 18 to 23 in the study population.

**Figure 1:**
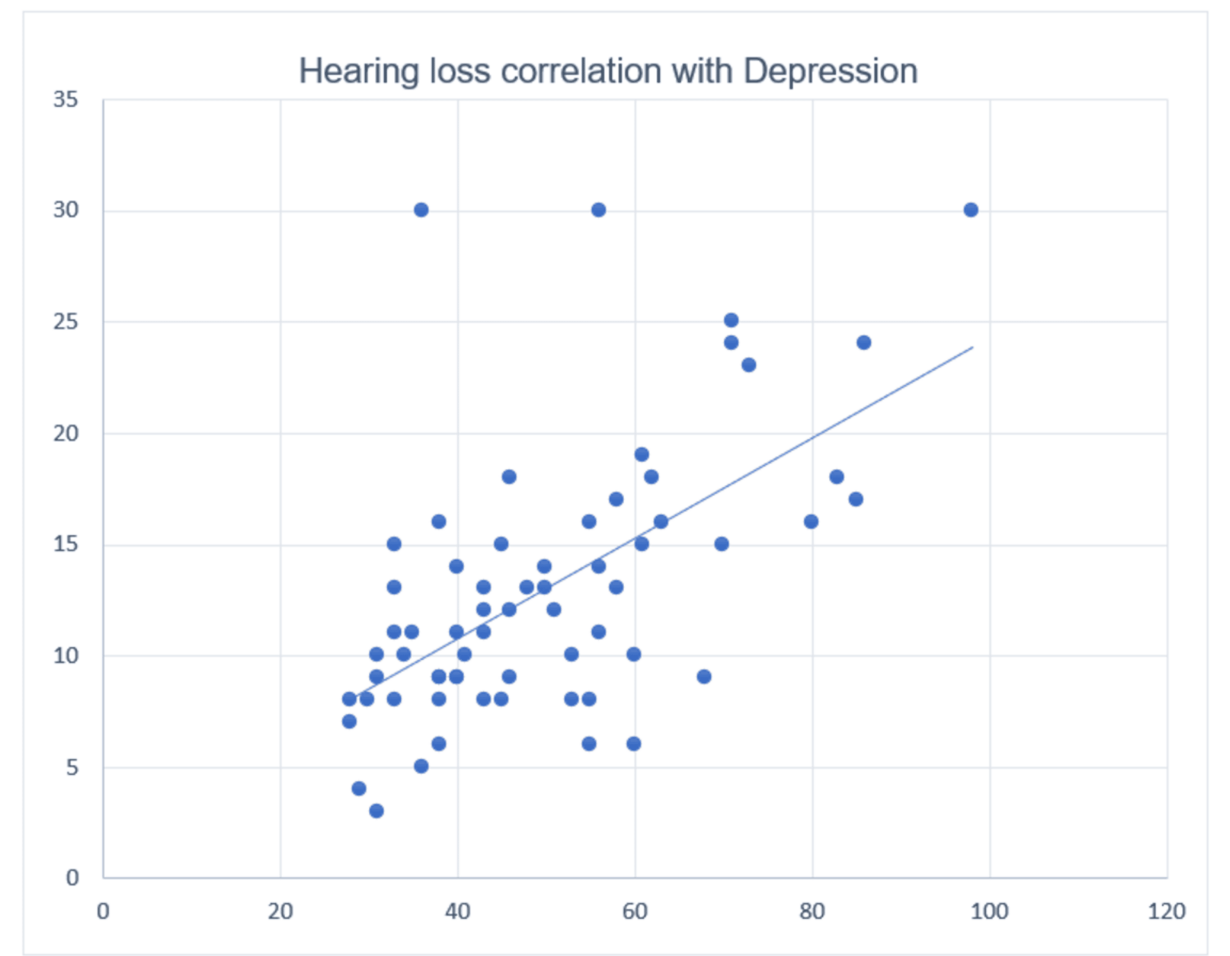
Pearson correlation between absolute values of hearing loss and depression scores x-axis: depression score, y-axis: hearing loss score

Figure 1 depicts the Pearson correlation between absolute values of hearing loss as noted from pure tone audiometry and depression scores taken from the Hamilton Depression Rating Scale. A significant correlation with r = 0.538 (p = 0.000) was observed.

On analysis, a significant association (p = 0.001) was observed between the grade of depressive symptoms and the degree of hearing loss. This implies that the greater the hearing loss, the more severe the signs of depression (Table 4).

**Table 4:** Degree of depressive symptomatology and degree of hearing loss

Depression grade	Degree of hearing loss				Total (N = 65)	χ^2^	p-value
	Mild (n = 24)	Moderate (n = 26)	Severe (n = 11)	Profound (n = 4)			
No	5	2	0	0	7	29.079	0.001
Mild	17	13	3	0	33		
Moderate	1	10	4	2	17		
Severe	1	1	4	2	8		

The mean score of hearing loss is maximum in severe depressive respondents. The difference among these four categories is significant (p = 0.009) according to the depression score. The mean speech reception threshold (SRT) scores were found to be maximum in severe depressive respondents. The difference among the four categories was significant (p = 0.013). The mean score of speech discrimination score (SDS) was also found to be maximum in the respondents with no depression. The difference among the four categories was significant (p = 0.000) (Table 5).

**Table 5:** Comparison of mean scores according to depression scores using one-way ANOVA and post hoc analysis Post hoc testing done using Tukey's HSD ANOVA: analysis of variance, SRT: speech reception threshold, SDS: speech discrimination score, HSD: honestly significant difference, a: none, b: mild, c: moderate, d: severe, NS: not significant, dB: decibels

Variable	Grade of depression				F value	p-value	Post hoc analysis
	None (n = 7) (a)	Mild (n = 33) (b)	Moderate (n = 17) (c)	Severe (n = 8) (d)			
Hearing loss	39.57 ± 12.843	43.88 ± 11.723	54.59 ± 15.565	69.00 ± 18.807	4.270	0.009	a versus d (significant), b versus d (significant)
SRT (dB)	45.00 ± 12.910	46.76 ± 11.992	55.88 ± 16.977	65.00 ± 13.784	3.874	0.013	a versus d (significant)
SDS	77.14 ± 9.063	76.21 ± 9.924	69.12 ± 13.138	62.50 ± 10.840	9.028	0.000	NS

Association with other variables

In analyzing the relationship between hearing loss and associated factors, no statistically significant difference was observed in hearing loss between individuals aged ≤70 years (45.42 dB) and those aged ≥70 years (52.58 dB) (p = 0.144). However, a significant difference was found in the speech reception threshold (SRT), with individuals aged ≥70 years demonstrating a higher mean SRT (54.48 dB) compared to those ≤70 years (46.67 dB) (p = 0.036). Similarly, the speech discrimination score (SDS) was significantly lower in individuals ≥70 years (70.15%) than in those ≤70 years (76.33%) (p = 0.002). In contrast, depression scores did not show a significant difference between the two age groups (p = 0.943).

Further analysis revealed no significant differences between male and female participants across any audiometric measures or depression scores: hearing loss (p = 0.612), SRT (p = 0.813), SDS (p = 0.490), and depression scores (p = 0.423). Similarly, tobacco use did not exhibit a statistically significant association with hearing loss (p = 0.436), SRT (p = 0.583), SDS (p = 0.404), or depression scores (p = 0.110). Likewise, no significant differences were found between alcohol consumers and non-consumers in terms of hearing loss (p = 0.588), SRT (p = 0.832), SDS (p = 0.263), or depression scores (p = 0.205).

Additionally, occupational noise exposure did not significantly impact any of the assessed variables. No statistically significant differences were observed in hearing loss (p = 0.597), SRT (p = 0.744), SDS (p = 0.222), or depression scores (p = 0.188) between individuals exposed to high versus low occupational noise levels.

The findings of this study highlight age as a critical factor in hearing function, particularly affecting speech reception and discrimination abilities. However, age did not appear to significantly influence depression scores. In contrast, factors such as gender, tobacco use, alcohol consumption, and occupational noise exposure showed no statistically significant associations with audiometric outcomes or depression. These results underscore the need for further research to explore the underlying mechanisms contributing to age-related changes in hearing and their potential impact on mental health.

## Discussion

Comparison with previous studies

The results of our study demonstrate a significant association between age-related hearing loss (ARHL) and depressive symptoms in the geriatric population of Uttarakhand, India. The Pearson correlation coefficient (r = 0.538, p = 0.00) indicates a moderate to strong positive correlation between the degree of hearing loss and depression scores. Additionally, the mean hearing loss score increases with the severity of depression, with statistically significant differences observed across depression categories (p = 0.009). These findings are consistent with previous studies by Teixeira et al. [8], Kim et al. [10], Lawrence et al. [11], and Kiely et al. [12], which have also reported a link between hearing loss and depression.

Several mechanisms may explain this association, including social isolation and loneliness resulting from hearing difficulties, as highlighted by Pronk et al. [13] and Stam et al. [14], both of whom identified these factors as significant contributors to depressive symptoms. Furthermore, Chi-square analysis revealed a significant association (p = 0.001) between the severity of depressive symptoms and the degree of hearing loss, confirming that as hearing loss worsens, so does the severity of depression.

While most studies support a positive correlation between hearing loss and depression, some report a positive but statistically insignificant correlation, while others find no association at all [15]. This variability in findings may be attributed to differences in study methodologies and diagnostic criteria. Notably, there remains a lack of research exploring the relationship between presbycusis and depression within the Indian context, highlighting the need for further investigation in this area.

In our study, 89.2% of the participants exhibited symptoms of depression. The majority of them (50.8%) experienced mild depressive symptoms, while 26.2% had moderate depression, and 12.3% suffered from severe depression. These findings align closely with the study by Nilforoush et al., which reported that 91.45% of individuals with ARHL also experienced depressive symptoms [1]. Additionally, our results are consistent with those of Gopinath et al. [16] and a multivariate study conducted in the United States [17].

Demographic analysis

Our analysis of hearing loss revealed that the mean hearing loss score was higher in individuals aged ≥70 years (52.58 ± 17.573) compared to those aged ≤70 years (45.42 ± 13.98). Although this difference was not statistically significant (p = 0.144, t = 1.875), it reflects a rising trend of hearing loss with increasing age. These findings align with those of Flatin et al. [18] and are further supported by a study conducted by Cruickshanks and Wichmann [19] in the United States, which reported a significant association between age and hearing loss.

Our study findings indicate no significant gender-based differences in audiometric parameters, including hearing loss, speech recognition threshold (SRT), and speech discrimination score (SDS), or depression scores among the study population (p = 0.612). These results highlight the importance of adopting a gender-neutral approach when assessing audiological and psychological outcomes in older adults.

Risk factor analysis

While several studies have reported a higher prevalence of presbycusis in men, our findings differ from those of Nolan [20], who identified a strong correlation between gender and ARHL. Similarly, studies by Flatin et al. [18] and Sousa et al. [21] also support a male predominance in hearing loss. One possible explanation is that men are more frequently exposed to occupational noise, a well-established risk factor for hearing loss [18,20]. Additionally, Flatin et al. propose that female hormones may have a protective effect on hearing, contributing to the observed gender disparity [18]. This hormonal influence is further supported by Wang and Puel, who suggest that estrogen may play a role in protecting against age-related hearing loss [22].

Our study found no statistically significant differences in audiometric parameters or depression scores between tobacco users and non-users, as well as alcohol consumers and non-consumers. These findings contradict some previous studies that have reported associations between tobacco use and hearing loss. For instance, a meta-analysis by Cruickshanks and Wichmann found a significant correlation between smoking and age-related hearing loss (ARHL), suggesting that tobacco consumption may accelerate the progression of hearing impairment [19]. However, other studies, such as those by Gopinath et al. [16] and Sousa et al. [21], have produced conflicting results, with some finding no significant association between smoking and hearing loss.

Interestingly, our study observed that tobacco users had a lower mean depression score compared to non-users, although this difference was not statistically significant. This contradicts existing research that links smoking to higher rates of depression. Studies such as that by Fluharty et al. have demonstrated a bidirectional relationship between smoking and depression, wherein smoking increases the risk of developing depression, and depression, in turn, increases the likelihood of smoking [23]. However, our findings suggest a different trend, warranting further investigation to understand the underlying mechanisms contributing to this discrepancy.

Additionally, our study found that alcohol consumers had slightly lower mean hearing loss values compared to non-consumers. This finding aligns with research by Fransen et al., which suggested that moderate alcohol intake might have a protective effect against hearing loss [24]. However, it contradicts a systematic analysis by Qian et al., which reported a positive association between alcohol consumption and hearing loss [25]. Other studies, such as that by Sousa et al., found no significant relationship between alcohol intake and hearing thresholds [21]. These inconsistencies highlight the need for further research to clarify the impact of tobacco and alcohol consumption on hearing and mental health.

In examining the severity of hearing loss across different sociodemographic groups, our study assessed the impact of occupational noise exposure, with 13 (20%) participants having worked in high-noise environments. However, our analysis did not reveal a significant correlation between occupational noise exposure and either hearing loss or depression. This lack of association may be due to the small sample size or the presence of confounding factors.

Our study further contributes to the growing body of evidence suggesting that ARHL may serve as a modifiable risk factor for depression in older adults [1,8]. Notably, like hearing loss, speech recognition threshold (SRT) scores also showed a significant increase with the severity of depression (p = 0.013). This indicates that individuals with more severe depressive symptoms may experience greater difficulties in speech perception, potentially due to cognitive and attentional impairments associated with depression [14].

Limitations of the study

This study provides valuable insights into the link between age-related hearing loss (ARHL) and depressive symptoms in the elderly population of Uttarakhand. However, its cross-sectional design limits the ability to determine causality. The relatively small sample size also affects the statistical power and generalizability of the findings. Additionally, the assessment of confounding factors was limited, which may influence the observed association. Future studies with larger, more diverse populations and longitudinal designs are needed to validate and expand on these results.

Future directions

Further longitudinal research is warranted to establish causality and develop evidence-based therapeutic strategies that integrate audiological and mental healthcare. Studies with larger sample sizes and diverse populations are needed to better understand the mechanisms underlying this association and identify optimal intervention strategies.

## Conclusions

Our study presents strong evidence of a significant link between age-related hearing loss (ARHL) and depressive symptoms among the elderly population in Uttarakhand. These findings underscore the importance of recognizing hearing loss not merely as a sensory issue but as a vital factor in the mental health of older adults.

Healthcare professionals should adopt a holistic approach to managing ARHL, one that goes beyond improving auditory function to also address its psychological and social impacts. Interventions such as hearing aids, cochlear implants, and other assistive technologies can play a crucial role in enhancing communication and reducing social isolation. Moreover, mental health support, including social engagement initiatives, cognitive behavioral therapy (CBT), and, where appropriate, pharmacological treatments such as antidepressants, can be effective in alleviating depression in this population. However, such treatments should be administered with careful consideration of age-related factors and comorbidities. Integrating auditory and mental healthcare is essential for improving the overall well-being and quality of life of older adults.

## Appendices

Case study form

General Information

Registration number:

Name:

Age/sex:

Occupation:

Address:

Religion:

Date of examination:

Present complaints:

History

A. Physical examination:

B. General examination:

(a) Body built:

(b) (i) Pallor:

(ii) Icterus:

(iii) Cyanosis:

(iv) Clubbing:

(v) Lymphadenopathy:

(vi) Edema:

(c) Vitals:

(i) Blood pressure:

(ii) Pulse:

(ii) Temperature:

(iii) Respiratory rate:

C. Relevant systemic examination:

1. Cardiovascular system:

2. Respiratory system:

3. Nervous system:

4. Locomotor system:

D. Ear examination:

(a) Ear: R/L

1. External ear:

(A) Pinna, size, shape, and level:

Any congenital abnormality:

(B) Pre-auricular region:

(C) Post-auricular region:

(D) External auditory canal (polyp or growth):

(E) Tympanic membrane:

1. Congestion:

2. Cone of light:

3. Retraction:

4. White deposits:

5. Mobility:

6. Perforation:

(i) Number:

(ii) Size:

(iii) Site:

(iv) Shape: 

(v) Margins:

7. Discharge:

(i) Quantity:

(ii) Quality:

(iii) Odor:

(iv) Serosanguinous or non-serosanguinous:

(v) Pulsatile or non-pulsatile:

(b) Tuning fork tests

(i) Rinne's:         Right:                                Left:

(ii) Webers:

(iii) ABC:            Right:                                 Left:

(a) Audiometric test

Pure tone audiometry

· Hearing loss type:     Right:                                Left:

· Hearing loss in decibels:    Right:                                Left:

· Grade of hearing loss according to WHO classification:     Right:                                Left:

Speech audiometry

· Speech reception threshold:     Right:                                Left:

· Speech discrimination score:     Right:                                Left:

(E) Assessment of depression after diagnosis:

(By Hamilton Depression Rating Scale-17 questionnaire)

Depression score:

Depression grading:
